# Gliotoxin Induces Cofilin Phosphorylation to Promote Actin Cytoskeleton Dynamics and Internalization of *Aspergillus fumigatus* Into Type II Human Pneumocyte Cells

**DOI:** 10.3389/fmicb.2019.01345

**Published:** 2019-06-18

**Authors:** Changjian Zhang, Fangyan Chen, Xiaoyu Liu, Xuelin Han, Yingsong Hu, Xueting Su, Yong Chen, Yansong Sun, Li Han

**Affiliations:** ^1^Chinese PLA Center for Disease Control and Prevention, Beijing, China; ^2^Academy of Military Medical Sciences, Beijing, China

**Keywords:** gliotoxin, cofilin, actin cytoskeleton rearrangement, *Aspergillus fumigatus*, human pneumocyte cells

## Abstract

*Aspergillus fumigatus* is able to internalize into lung epithelial cells to escape from immune attack for further dissemination. We previously reported that gliotoxin, a major mycotoxin of *A. fumigatus*, promotes this internalization; however, the mechanism remained unclear. Here, we report that gliotoxin is able to induce cofilin phosphorylation in A549 type II human pneumocytes. Either too high or too low a level of cofilin phosphorylation blocked the gliotoxin-induced actin cytoskeleton rearrangement and *A. fumigatus* internalization. LIM domain kinase 1 (LIMK1) and its upstream small GTPases (Cdc42 and RhoA, but not Rac1) predominantly mediated the gliotoxin-induced cofilin phosphorylation and *A. fumigatus* internalization. Simultaneously, gliotoxin significantly stimulated an increase in cAMP; however, adding an antagonist of PKA did not block gliotoxin-induced *A. fumigatus* internalization. *In vivo*, exogenous gliotoxin helped gliotoxin synthesis deficient strain *gliPΔ* invade into the lung tissue and the lung fungal burden increased markedly in immunosuppressed mice. In conclusion, these data revealed a novel role of gliotoxin in inducing cofilin phosphorylation mostly through the Cdc42/RhoA-LIMK1 signaling pathway to promote actin cytoskeleton rearrangement and internalization of *A. fumigatus* into type II human pneumocytes.

## Introduction

As an opportunistic fungal pathogen widely distributed in the environment, including the soil, decaying vegetation, and compost heaps (Gugnani, 2003; Warris et al., 2003; Segal, 2009), *Aspergillus fumigatus* releases numerous air-borne conidia that can reach the pulmonary alveoli of human hosts (Gomez et al., 2010) and cause invasive aspergillosis (IA), a devastating disease with a mortality rate of nearly 90% in immunocompromised populations (Lin et al., 2001). It is well known that *A. fumigatus* has dynamic interactions with host immune cells, including macrophages, neutrophils, and mononuclear cells in the lungs, through different polysaccharides on its cell surface and several secretory factors, such as toxins, proteinases, and phospholipases (Abad et al., 2010; van de Veerdonk et al., 2017). Recently, alveolar epithelial cells, which are usually considered as an anatomic barrier, have been found to play an intriguing role in the development of aspergillosis. Apart from the fact that they release various proinflammatory factors in response to *A. fumigatus* infection, alveolar epithelial cells can be invaded by *A. fumigatus* conidia, serving as a safe haven for evasion from immune attack and helping conidia dissemination throughout the host (Beisswenger et al., 2012). The process of *A. fumigatus* internalization into epithelial cells depends on the actin cytoskeleton dynamic assembly, which induces the host cell membrane invagination and wraping up the conidia (Wasylnka and Moore, 2003; Botterel et al., 2008; Croft et al., 2016). Previous studies showed that β-1,3-glucan (Han et al., 2011) and PacC (Bertuzzi et al., 2014), a pH sensitive transcription factor produced by *Aspergillus*, induce the swollen conidia of *A. fumigatus* internalization into host cells depending on the presence of Dectin-1, an important lectin-like pattern recognition receptor in anti-fungal immunity (Han et al., 2011; Wiesner and Klein, 2017). Many other studies have shown that silencing of E-cadherin expression also decreased the internalization of conidia into A549 cells (Xu et al., 2012; Yan et al., 2015). However, the exact regulatory mechanism of this complex internalization process is still under investigation.

Gliotoxin is a secondary metabolite, the most abundant mycotoxin secreted by *A. fumigatus* (Gardiner et al., 2005; Stanzani et al., 2005). It is a member of the epipolythiodioxopiperazine family of fungal toxins, which carries an internal disulfide bridge that is essential for its function (Scharf et al., 2012; Schlam et al., 2016). Gliotoxin has a wide range of immunomodulatory capabilities, inhibiting the phagocytosis of macrophages and the immune functions of other immune cells (Nishida et al., 2005; Peterson et al., 2010; Schlam et al., 2016). Gliotoxin is thought to indirectly inhibit inducible NF-κB activity by preventing the proteosomal degradation of its inhibitor, IκB, which consequently induces host-cell apoptosis (Kroll et al., 1999; Stanzani et al., 2005; Geissler et al., 2013; Scharf et al., 2016). Gliotoxin inhibits phagocytosis and affects the organization of the actin cytoskeleton via distinct signaling pathways in human neutrophils; the anti-phagocytic property of gliotoxin is arachidonic acid-dependent and cAMP-independent, while the gliotoxin-induced actin cytoskeleton rearrangement is cAMP-dependent and arachidonic acid-independent (Comera et al., 2007). It has been demonstrated that gliotoxin is closely associated with the development of IA (Gardiner et al., 2005), and gliotoxin production contributes to the virulence of *A. fumigatus* in mice immunosuppressed with hydrocortisone or cortisone acetate (Sugui et al., 2007; Spikes et al., 2008).

Interestingly, gliotoxin has been detected not only in the blood and alveolar lavage fluid of patients with IA, but also in the moist environments, including surfaces of objects contaminated with the fungus and even in the air or ventilation system (Peterson et al., 2010; Geissler et al., 2013). In one study, around 0.43–1.12 pg/mg gliotoxin in building material and 400 pg/cm^2^ in the dust have been detected (Thrasher and Crawley, 2009). All these data hinted that gliotoxin might be inhaled into the alveoli of lung. However, the influence of gliotoxin on lung epithelial cells and on the development of aspergillosis, especially in the early phase of infection of the alveolar niche is still unknown.

We previously reported that gliotoxin induced obvious morphological changes in A549 type II human pneumocytes but had no effect on cell viability at concentrations below 100 ng/ml (Jia et al., 2014). Intriguingly, 50 ng/ml gliotoxin enhanced the internalization of *A. fumigatus* conidia by A549 cells but inhibited the phagocytosis of J774 macrophages in a dose-dependent manner (Jia et al., 2014). Meanwhile, we showed that cofilin, a member of the actin depolymerizing factor family (Bernstein and Bamburg, 2010) was involved in regulating the internalization of *A. fumigatus* into lung epithelial cells through the RhoA-ROCK-LIM kinase pathway, which was interestingly not related to dectin-1 receptor and its ligand, β-1,3-glucan (Bao et al., 2015).

As a critical modulator of actin dynamics in mammalian cells (Taylor et al., 2011), cofilin functions to depolymerize the filamentous actin. When the serine residue at its N-terminus of cofilin is phosphorylated by the upstream kinases, like LIM kinase (LIMK), this phosphorylated cofilin (p-cofilin) loses the depolymerization activity. The p-cofilin can be recycled with dephosphorylation by phosphoric acid hydrolase, like Slingshot to recover its activity. In response to various intracellular and extracellular stimuli, two major upstream signals in mammalian cells regulate the cofilin activity, one is small Rho GTPases, like Rho1/Cdc42/Rac1; the other is cAMP/PKA (Lee et al., 2015; Romarowski et al., 2015; Hu et al., 2016). In addition, the ratio of cofilin/actin within a cell is known to determine whether the overall effect of cofilin is primarily a severing action or one that promotes actin polymerization (Andrianantoandro and Pollard, 2006). Taken together, it is intriguing to speculate that gliotoxin may regulate cofilin activity, and consequently induce internalization of *A. fumigatus* into epithelial cells during infection.

In this study we examined the role of gliotoxin in the internalization of *A. fumigatus* spores both by *in vivo* experiments in mice and *in vitro* using the human alveolar epithelial cell line A549. In the *in viro* experiments we focused on the effects of gliotoxin on the cellular pathways leading to cofilin phosphorylation and on the role of cofilin in the internalization event.

## Materials and Methods

### Chemical Reagents, Antibodies, siRNAs, and Plasmids

Gliotoxin (#ab142437) was purchased from Abcam, (United Kingdom); BMS-5 (#SYN-1024) was purchased from Synkinase; Y27632 (#Y0503), pCT-cAMP (#C3912), Rp-cAMP (#A165), latrunculin A (#L5163), cucurbitacin E (#SML0577), and LY294002 (#S1105) were purchased from Sigma-Aldrich, (United States); ML141 (#s7686) and EHOP-016 (#s7319) were purchased from Selleckchem, (United States). Rabbit monoclonal anti-phospho-cofilin (#3313), rabbit monoclonal anti-LIMK1 (#3842S), and rabbit monoclonal anti-phospho-LIMK1 (#3841S) antibodies were purchased from Cell Signaling Technology (United States). Mouse monoclonal anti-β-actin (#sc-47778) and rabbit polyclonal anti-cofilin (#sc-33779) antibodies were obtained from Santa Cruz Biotechnology (United States). HPR-conjugated goat anti-mouse IgG and HRP-conjugated goat anti-rabbit IgG antibodies were obtained from ZSGB-BIO (China). siRNA universal negative control and siRNA-cofilin (siRNA ID: SASI_ Hs01_00078353) were designed and chemically synthesized by Sigma-Aldrich, (United States). The pCMV/pCAG-LifeAct plasmids were purchased from ibidi (Germany). Plasmids encoding wild-type cofilin, non-phosphorylatable S3A cofilin, and phosphorylated S3D cofilin (each subcloned into pcDL-SRα) were preserved in our laboratory. Plasmids encoding wild-type cofilin, non-phosphorylatable S3A cofilin, and phosphorylated S3D cofilin conjugated to green fluorescent protein (GFP; subcloned into pEGFP C1) were constructed in this study.

### Cell Line and Fungal Strains

The type II human alveolar epithelial cell line A549 was obtained from the ATCC and was cultured at 37°C under 5% CO_2_ in RPMI 1640 supplemented with 10% heat-inactivated fetal calf serum (FBS), 100 U/ml streptomycin, and 100 U/ml penicillin. *A. fumigatus* wild-type strain B5233, its mutant strain *gliPΔ* strain (deletion of the peptide synthetase *gliP*) and the *gliP* reconstituted strain *gliPR* were kindly gifted by Dr. K. J. Kwon-Chung (National Institute of Health, Bethesda, MD, United States). *A. fumigatus* ATCC13073 constitutively expressing green fluorescent protein was generously provided by Dr. Margo Moore (Simon Fraser University, Burnaby, BC, Canada). *A. fumigatus* ku80 constitutively expressing red fluorescent protein mCherry (subcloned into pJW103) was constructed in our laboratory. Conidia were propagated on Sabouraud dextrose agar (SDA) for 5–8 days at 37°C and were collected with 0.1% Tween-20 (PBST). The conidia were passed through a filter (40 μm) to remove hyphal fragments and enumerated using a hemocytometer. The culture filtrates of *A. fumigatus* were prepared by inoculating 1 × 10^7^ conidia in 50 ml of RPMI 1640 and incubating them for 48 h at 37°C and 5% CO_2_ as shown in previous study (Sugui et al., 2007).

### Immunofluorescence Analysis

A549 cells were seeded onto coverslips in 24-well plates (1 × 10^4^ cells/well) and grown for 24 h. After treatment with the indicated pharmacological agents, the cells were fixed in ice-cold 4% paraformaldehyde/PBS at room temperature for 15 min. Then, the cells were permeabilized with 0.1% Triton X-100/PBS at 4°C for 10 min. The samples were blocked in 5% bovine serum albumin (BSA) at room temperature for 30 min, and incubated with primary antibodies (cofilin, p-cofilin) for 2 h and then with fluorochrome-conjugated secondary antibodies (green) for 1 h. Additionally, 5 μM tetramethyl rhodamine phalloidin (Invitrogen R415, 1:50) was added for 20 min, 0.3 μM Fluorescent Deoxyribonuclease I conjugates was added for 20 min and 1 μg/ml DAPI (4′,6-diamidino-2-phenylindole) was added for 5 min to label filamentous actin (F-actin, red), globular actin (G-actin, green), and nuclei (blue), respectively. The preparations were observed under a fluorescence microscope (Olympus BX51; magnification, 400×) equipped with an Olympus DP71 camera, or a laser confocal microscope (Olympus IX83). The images were processed using Image Pro Express 6.0 (Media Cybernetics, Rockville, MD, United States). The F-actin/G-actin ratio in cells was quantitatively analyzed with at least ten images by examining the size and light intensity of fluorescent areas with the ImageJ software. Experiments were performed in three independent experiments.

### Live-Cell Imaging

A549 cells were seeded in laser confocal cell culture dish (2 × 10^5^ cells/dish) and grown for 24 h. Then, the cells were transfected with the pCMV/pCAG-LifeAct plasmids, an actin marker for the visualization of F-actin (red) in living cells, or plasmids encoding cofilin-wild type-GFP for the visualization of cofilin (green) in living cells. After transfection 48 h, the conidia of *A. fumigatus* ATCC13073 (green) and Ku80 (red) were added into the cells, respectively. And the cells were immediately monitored by Time-lapse spinning disk confocal microscope (Perkin Elmer UltraVIEW VOX, United States) using 40× objective. Live cells were imaged in a temperature-controlled chamber (37°C) at 5% CO_2_ at 1 frame every 2 min. Dual-color videos were acquired as consecutive green–red images. Experiments were performed in three independent experiments.

### Western Blotting

Cells were seeded in a 6-well plate (8 × 10^5^ cells/well) and cultured overnight. After exposure to gliotoxin with or without the indicated pharmacological agents pretreatment, 20 μM BMS-5 (LIMK inhibitor), 20 μM Y27632 (ROCK inhibitor), 50 μM ML141 (Cdc42 inhibitor) or 0.5 μg/ml Rho inhibitor I (RhoA inhibitor) for 1 h, 2 μM EHOP-016 (Rac1 inhibitor) for 24 h, 100 μM pCPT-cAMP (PKA agonist) or 100 μM Rp-cAMP (PKA antagonist) for 30 min, the cells were washed with cold PBS. The cells were lysed in cold lysis buffer containing 150 μM NaCl, 5% (w/v) 1 M Tris–HCl (pH 7.5), 0.5 μM EDTA.Na_2_, and 1% (v/v) NP40. After centrifugation (13000 rpm, 20 min), the supernatant was collected as the total cellular protein extract. Equal amounts of protein were separated by sodium dodecyl sulfate polyacrylamide gel electrophoresis (5 to 12% gradient) and then transferred to a polyvinylidene fluoride membrane. The membrane was incubated with the indicated primary antibodies at room temperature for 2 h or at 4°C overnight, and subsequently with the HRP-conjugated secondary antibody at room temperature for 1 h. The proteins were visualized using enhanced chemiluminescence (Santa Cruz Biotechnology). Band intensity was quantified using ImageJ software and was normalized to that of β-actin or GAPDH. Brightness and contrast were adjusted evenly across the entire image. Experiments were performed in three independent experiments.

### Measurement of Intracellular cAMP

Cells were seeded in a 6-well plate (8 × 10^5^ cells/well) and cultured overnight. Medium was then changed to serum-free medium and cells were incu- treated with 50 ng/ml gliotoxin for the indicated periods. Culture supernatants were aspirated and the cells were washed in cold PBS three times. Then the intracellular cAMP concentrations were determined by ELISA according to the manufacturer (R&D company, #KGE002B). Experiments were performed in three independent experiments with three individual replicates.

### Analysis of *A. fumigatus* Internalization

The nystatin protection method was used to determine the internalization of *A. fumigatus* into A549 cells. The cells were seeded in 96-well plates (2 × 10^4^ cells/well) 24 h prior to the experiment and then pretreated with pharmacological agents at 37°C for the indicated times in 5% CO_2_. According to the conidial germination time in cell culture medium, the cells were incubated with *A. fumigatus* resting conidia at multiplicity of infection [MOI] = 20 for 6 h at 37°C in 5% CO_2_ (Han et al., 2011; Jia et al., 2014). Then, the cells were washed with PBST and incubated with nystatin 100 μl/well (30 μg/ml) in medium for 4 h at 37°C. The monolayers were removed and the wells were washed with PBST again. Then, the cells were lysed by incubation in 0.25% Triton X-100 for 15 min. The released conidia were diluted, plated onto SDA (3 replicate plates/well), and incubated at 37°C for 18 h. The colonies were counted to determine the total intracellular conidia. The internalization capacity is expressed as a percentage of the initial inoculum. Experiments were performed in three independent experiments with three individual replicates.

### Mouse Infections

This study was carried out in accordance with the recommendations of Management of Laboratory Animal, Laboratory Animal Welfare and Ethics Committee, Academy of Military Medical Sciences, China. The protocol was approved by the Laboratory Animal Welfare and Ethics Committee (IACUC-13-2016-002), Academy Military Medical Sciences, China. C57BL/6 mice were obtained from Animal Center of Academy of Military Medical Sciences. The mouse model of invasive pulmonary aspergillosis was established using a protocol described by Sugui et al. (2007), which involves immunosuppression with steroids, with modification. Ten- to twelve-week-old male C57BL/6 mice were treated with hydrocortisone acetate (5 mg/mouse, administered subcutaneously) every other day, beginning on day –4 relative to infection and ending on day +4, for a total of 5 doses. For inoculation, the mice were anesthetized with isoflurane, after which 5 × 10^6^ conidia in 25 μl of PBS containing 0.1% Tween-20 was instilled intranasally. The mice were infected with *A. fumigatus* alone or with *A. fumigatus* mixed with exogenous 50 ng/ml gliotoxin. For survival analysis, the mice were weighed every day after infection. In the majority of cases, the end-point for survival experimentation was death. At the relevant time-points post-infection, mice were sacrificed and lungs tissues were weighed, homogenized, and then serially diluted onto Sabouraud agar plates for incubation at 37°C. After 20 h, the number of colonies in lung tissues (number of CFU/g) was also counted and calculated. For histopathological examination, the lung tissue sections obtained from mice from each group at 72 h post-infection were dissected, fixed in 10% (vol/vol) formaldehyde, and stained with hematoxylin and eosin (HE) and periodic acid-Schiff (PAS). Images were taken using a BX51 microscope (Olympus) with an Olympus DP71 camera using brightfield illumination at 200× or 400× magnification. These images were later processed using Image Pro Express 6.0 (Media Cybernetics Inc., Rockville, MD, United States).

### Determination of Fungal DNA From Infected Lungs by Quantitative RT-PCR

A quantitative RT- PCR approach was selected to determine the fungal burden based on a previous study (Ibrahim-Granet et al., 2010). In brief, the complete lung from each mouse was frozen in liquid nitrogen and ground to a fine powder. Approximately 50 mg of each powdered lung was used for DNA extraction. After ethanol precipitation, the amount of DNA extracted from the lung tissues was quantified by a U-Drop spectrophotometer. All samples were diluted to 100 ng/μl and quantified again to confirm the DNA concentration of each sample. The standard curve on genomic DNA from *A. fumigatus* was generated from three technical replicates, whereby each replicate contained 6 dilutions in the range between 100 and 3.125 ng per reaction (stability index of standard curve = 0.99). qRT-PCR was performed using SYBR *Premix Ex Taq* (Tli RNaseH Plus) (TaKaRa, Cat. # RR420A, Japan) according to the manufacturer’s protocols and reaction was performed by Roche Light Cycler® 96 SW 1.1 Detection System. The amplification program consisted of an initial denaturation at 95°C for 10 min followed by 40 cycles with denaturation for 15 s at 95°C, annealing for 30 s at 54°C, and amplification for 30 s at 72°C. All data points from each sample were used for calculation of the amount of fungal DNA per μg of total DNA. Experiments were performed in three independent replicates.

### Statistical Analysis

Data are presented as the mean ± standard error of the mean (SEM) of three independent experiments. The significance of differences was assessed by Log-Rank analysis (for comparative survival), one-way ANOVA followed by Tukey *post hoc* test or by Dunnett *post hoc* test, or unpaired Student’s *t*-tests with a 95% confidence interval, using GraphPad Prism software. ^*^p < 0.05, ^∗∗^*p* < 0.01, and ^∗∗∗^*p* < 0.001.

## Results

### Cofilin Is Involved in Gliotoxin-Induced Actin Cytoskeleton Arrangement in Lung Epithelial Cells

**FIGURE 1:**
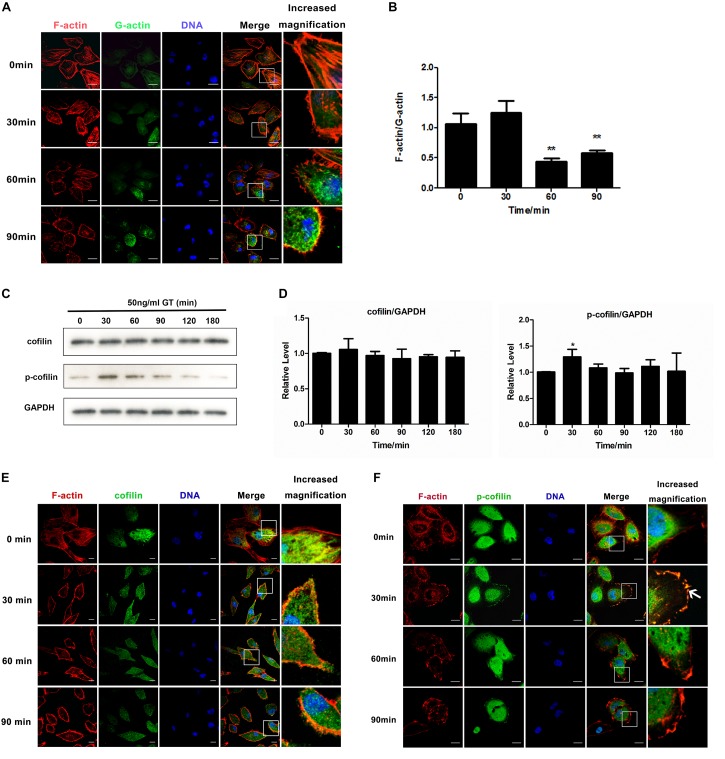
Cofilin is involved in gliotoxin-induced actin cytoskeleton arrangement in lung epithelial cells. A549 cells were treated with 50 ng/ml gliotoxin for the indicated periods. **(A)** The distribution of F-actin and G-actin. Red, F-actin; Green, G-actin; Blue, nuclei. **(B)** The F-actin/G-actin ratio. **(C)** The expression of cofilin and the phosphorylation level of cofilin (p-cofilin) were measured by western blotting. **(D)** The relative levels of cofilin and p-cofilin to GAPDH were analyzed using Image J2 software. **(E,F)** Distribution of cofilin and p-cofilin in cells. Red, F-actin; Green, cofilin or p-cofilin; Blue, nuclei. The magnified image is of the white-boxed area in the merged image. White arrows point to the clustering of p-cofilin and the colocalization of F-actin and p-cofilin at the cell cortex. Experiments were performed in three independent experiments with three individual replicates. Images shown are representative of the independent experiments. Statistically significant differences were determined using one-way ANOVA followed by Tukey *post hoc* test. ^∗∗^*p* < 0.01, ^*^p < 0.05. Scale bar = 20 μm.

Our previous study showed that 50 ng/ml gliotoxin markedly affected cell morphology, but not viability of lung epithelial cells (Jia et al., 2014). In this study, we firstly investigated the distribution of F-actin and monomeric, globular actin (G-actin) under stimulation of gliotoxin in A549 cells. With the prolongation of gliotoxin treatment, the level of F-actin in the cytoplasm decrease after 60 min with an obvious concomitant increase of cytoplasmic G-actin (Figure 1A). In addition, quantitative analysis also indicated that the ratio of F-actin to G-actin in the whole cells decreased significantly by gliotoxin treatment (Figure 1B).

As gliotoxin can induce apoptosis in different types of cells, including macrophages, neutrophils, epithelial cells, and fibroblasts (DeWitte-Orr and Bols, 2005; Stanzani et al., 2005), we checked apoptosis in lung epithelial cells after exposure to gliotoxin by flow cytometry. It was not found that 50 ng/ml gliotoxin can induce apoptosis or influence cell viability over the first 2 h (Supplementary Figure S1). This indicated that the obvious actin cytoskeleton rearrangement in lung epithelial cells induced by 50 ng/ml gliotoxin was not associated with apoptosis or cell death.

Next, we asked how gliotoxin induces actin cytoskeleton rearrangement in lung epithelial cells. It was generally accepted that *A. fumigatus* internalization into lung epithelial cells is an actin dynamic process and we also reported that cofilin is involved in regulation of *A. fumigatus* internalization in epithelial cells (Bao et al., 2015). Here in this study we provided for the first time the evidence by live cell imaging. As shown in Supplementary Figure S2 and Supplementary Movie S1, it was clearly illustrated that the GFP-labeled conidia of *A. fumigatus* ATCC13073 invaded into A549 cells along with a RFP-labeled actin cap at the initial invasion site (Time 1:33:54, left panel, white arrow or Time 3:09:54, right panel, white arrow); and later the RFP-labeled actin was enriched around an internalized conidium, forming a red ring (Time 1:55:54, left panel or Time 3:45:52, right panel). In Supplementary Figure S3 and Supplementary Movie S2, the conidium with red fluorescence were added into A549 cells expressing GFP-cofilin and it was found that at the time point 3 h 46 min (03:46) or 4 h 46 min (04:46), a yellow ring around red conidia was formed by the merge of the red conidia and green color of GFP-cofilin together, which indicated the colocalization of cofilin with conidia. Similarly, in the video some other yellow rings were also found at the time points around 04:56, 05:28, 05:36, 06:20 and 06:42, 07:02, 07:34 around different conidium, respectively. These data demonstrated that cofilin is involved in the regulation on the uptake of conidia into lung epithelial cells. Thus, we examined whether gliotoxin is able to regulate cofilin activity. As shown in Figures 1C,D, the expression of cofilin was unchanged, whereas cofilin with a phosphorylated serine residue at its N-terminus (p-cofilin) significantly increased, peaked at 30 min post-stimulation, and then gradually returned to the basal level. This effect of gliotoxin on cofilin phosphorylation was also confirmed in Beas2B airway epithelial cells (Supplementary Figure S4A). Since the gliotoxin is the major secreted mycotoxin of *A. fumigatus*, we exposed the A549 cells to the culture supernatants of different strains, WT, *glipΔ*, and *glipR* and compared the cofilin phosphorylation in A549 cells. It was found that the cofilin phosphorylation induced by the culture supernatant of *glipΔ* is much lower than other two groups at 30 min post-treatment (Supplementary Figure S4B). Further, the alteration of the localization of both non-phosphorylated cofilin and p-cofilin in A549 cells upon gliotoxin treatment was observed by confocal microscopy. As illustrated in Figure 1E, cofilin was distributed throughout the cytoplasm and colocalized with F-actin at some lung epithelial cell cortexes (Figure 1E), which was hardly altered by gliotoxin treatment. In contrast, as shown in Figure 1F, the distribution of p-cofilin was significantly changed by gliotoxin stimulation at 30 min post-stimulation. P-cofilin was distributed more strongly at the cell cortex as compared to untreated cells, and also colocalized with F-actin. Taken together, both western blot and immunofluorescence data showed that p-cofilin level and its cell distribution return to the untreated status after 90 min. These results suggested that gliotoxin was able to induce cofilin phosphorylation and might also have an impact on the intracellular distribution of p-cofilin.

**FIGURE 2:**
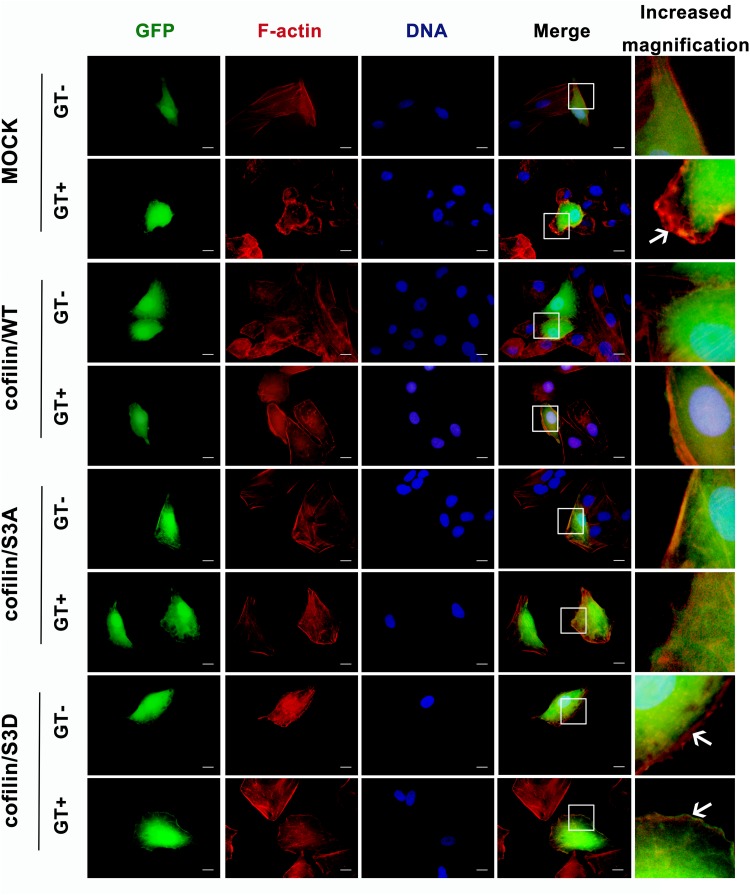
Effect of cofilin on gliotoxin-induced actin cytoskeleton rearrangement in lung epithelial cells. A549 cells were transfected with vector expressing green fluorescent protein (GFP) (control), GFP-tagged cofilin/WT, GFP-tagged cofilin/S3A, or GFP-tagged cofilin/S3D plasmid. After 48 h, the cells were treated with 50 ng/ml gliotoxin for 30 min. The distribution of F-actin and the different cofilin proteins was monitored by fluorescence microscopy. Red, F-actin; Green, GFP-tagged protein. Experiments were performed in three independent experiments with three individual replicates. Images shown are representative of the independent experiments. The white arrow indicates the actin cytoskeleton rearrangement. Scale bar = 20 μm.

To investigate the effect of cofilin phosphorylation on gliotoxin-induced actin cytoskeleton rearrangement in lung epithelial cells, three plasmids, expressing wild-type cofilin (cofilin/WT), constitutively active non-phosphorylatable S3A cofilin (cofilin/S3A), or inactive phosphomimic S3D cofilin (cofilin/S3D), labeled with green fluorescent protein (GFP), were constructed (Supplementary Figures S5A,B). As illustrated in Figure 2, in the absence of gliotoxin, transfection with any of the three plasmids did not significantly alter the basal actin cytoskeleton of A549 cells as compared to cells transfected with empty plasmid. Intriguingly, the obvious actin cytoskeleton rearrangement with production of ruffles or lamellipodia induced by gliotoxin in the Mock-infected control group (control group, GT panel, indicated by white arrow) was apparently hindered by either overexpression of wild-type cofilin, constitutively active S3A cofilin, or inactive phosphor-mimic S3D cofilin. Especially, the separation of cofilin from F-actin induced by gliotoxin at cell edge was observed in cells expressing S3D cofilin (Figure 2, cofilin/S3D group, GT panel, white arrow). These results indicated that either too high or too low phosphorylation of cofilin, which is closely related with its depolymerizing activity, may deteriorate the gliotoxin-induced actin cytoskeleton rearrangement in lung epithelial cells. A normal and balanced cofilin phosphorylation seems to be indispensable to this process.

### Gliotoxin-Induced Actin Cytoskeleton Rearrangement Is Modulated by the Cdc42/RhoA-LIMK1-Cofilin Signaling Pathway

**FIGURE 3:**
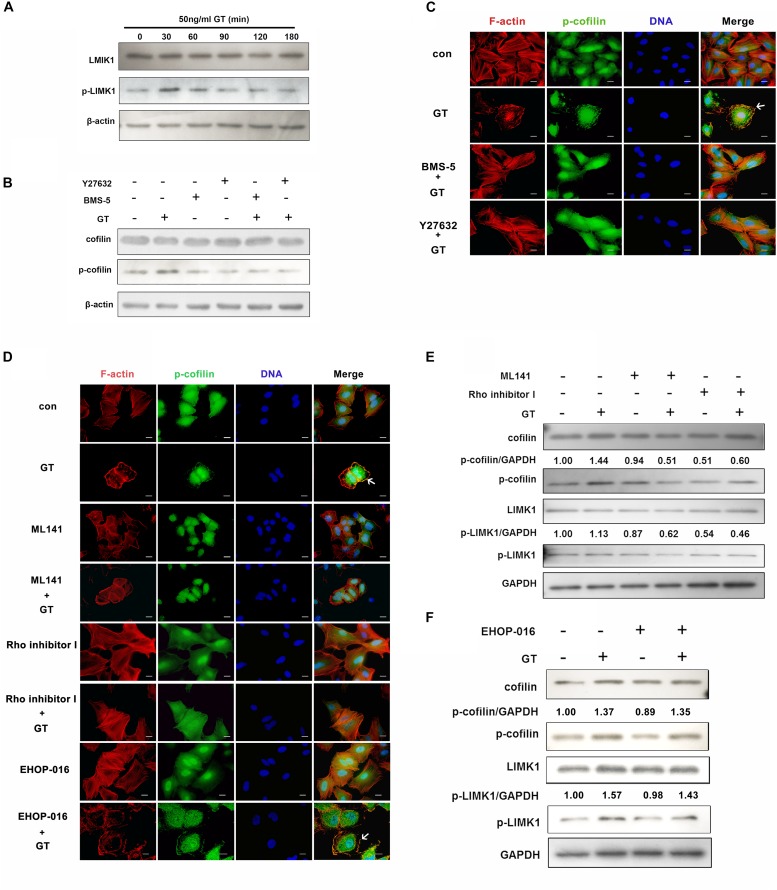
Gliotoxin-induced actin cytoskeleton rearrangement is modulated by Cdc42/RhoA-LIMK1-cofilin signaling pathway. **(A)** The expression and the phosphorylation level of LIMK1 in A549 cells treated with gliotoxin for the indicated periods. **(B–F)** A549 cells were pretreated with the indicated pharmacological agents, followed by treatment with 50 ng/ml gliotoxin for 30 min. The expression and the phosphorylation level of cofilin and LIMK1 were determined by western blotting; The distribution of F-actin and p-cofilin was monitored by fluorescence microscopy. Red, F-actin; Green, p-cofilin; Blue, nuclei. Experiments were performed in three independent experiments. Images shown are representative of the independent experiments. The white arrow indicates the actin cytoskeleton rearrangement and the colocalization of F-actin and p-cofilin at the cell cortex. Scale bar = 20 μm.

It is well known that cofilin can be phosphorylated by its upstream regulator, LIMK1, the most extensively studied subtype of LIM kinases (Wang and Townes-Anderson, 2016). Thus, the effect of gliotoxin on LIMK1 in lung epithelial cells was tested. Compared to the control group, the expression of LIMK1 protein was not changed, whereas the phosphorylation level of LIMK1 was increased after 30 min of treatment with gliotoxin, and then gradually decreased to the basal level (Figure 3A). The influence of LIMK1 on gliotoxin-induced cofilin phosphorylation was also analyzed. To this end, two inhibitors were used: BMS-5, a specific direct inhibitor of LIMK, and Y27632, a specific inhibitor of Rho kinase (ROCK), a putative upstream kinase of LIMK1. As shown in Figure 3B, gliotoxin-induced cofilin phosphorylation was significantly inhibited by either BMS-5 or Y27632. Meanwhile, the F-actin aggregation and p-cofilin redistribution induced by gliotoxin in A549 cells were also blocked by pretreatment with these two inhibitors, as indicated by immunofluorescence microscopy (Figure 3C). These data suggested that LIMK1 might mediate gliotoxin-induced actin cytoskeleton rearrangement in lung epithelial cells through cofilin phosphorylation.

As actin cytoskeleton rearrangement in cells is mainly modulated by the small Rho-family GTPases, including Rac1, RhoA and Cdc42, which specifically control the formation of lamellipodia, stress fibers, and filopodia, respectively (van Buul and Timmerman, 2016), the roles of these three small GTPases were further investigated. As illustrated in Figure 3D, compared to the control group, the inhibition of Cdc42 by its specific inhibitor, ML141, significantly blocked gliotoxin-induced actin cytoskeleton rearrangement in A549 cells, but did not strongly affect cell morphology in the absence of gliotoxin (Figure 3D). Rho inhibitor I, which specifically inhibits RhoA protein, had a similar effect on the gliotoxin-induced actin dynamic. However, the Rac1 inhibitor EHOP-016 was not capable of blocking the gliotoxin-induced actin cytoskeleton rearrangement and the clustering of p-cofilin at the cortex (Figure 3D, EHOP-016+GT group, white arrow). In accordance with these immunofluorescence results, ML141 and Rho inhibitor I (Figure 3E), but not EHOP-016 (Figure 3F), obviously hindered gliotoxin-induced phosphorylation of cofilin and LIMK1. All the inhibitors had little impact on cell viability (Supplementary Figure S6). Taken together, the results indicated that both Cdc42 and RhoA are involved in regulating gliotoxin-induced actin cytoskeleton rearrangement through LIMK1 and cofilin phosphorylation in lung epithelial cells.

### cAMP/PKA Signaling Is Also Involved in Gliotoxin-Induced Actin Cytoskeleton Rearrangement in Lung Epithelial Cells

**FIGURE 4:**
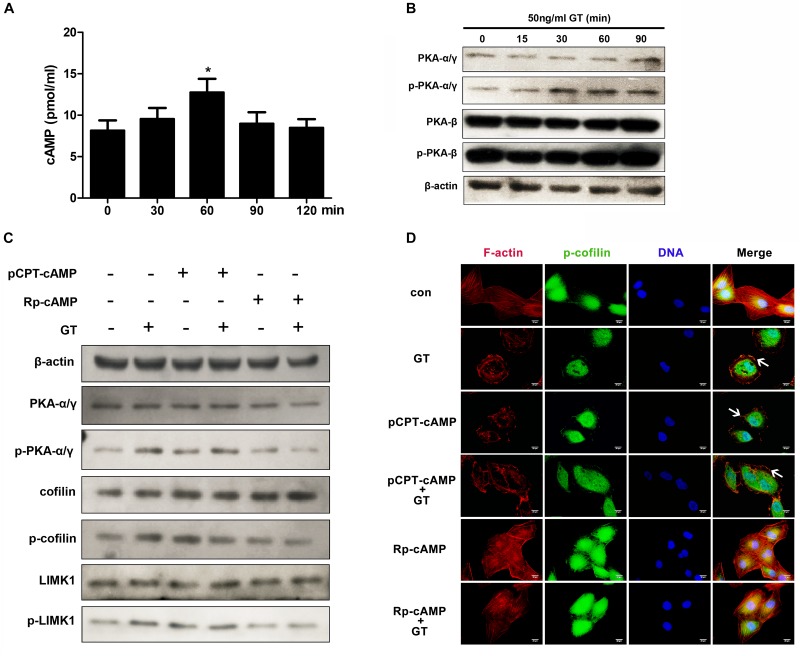
cAMP/PKA signaling is involved in gliotoxin-induced rearrangement of actin cytoskeleton. **(A)** The intracellular concentration of cAMP in A549 cells treated with gliotoxin for the indicated periods. **(B)** The intracellular expression and phosphorylation of both PKA_α/γ_ and PKA_β_ in A549 cells treated with gliotoxin for the indicated periods. **(C,D)** A549 cells were pretreated with the indicated pharmacological agents, followed by treatment with 50 ng/ml gliotoxin for 30 min. The expression and phosphorylation of PKA_α/γ_, cofilin, and LIMK1 were detected by western blotting; The distribution of F-actin and p-cofilin was monitored by fluorescence microscopy. Red, F-actin; Green, p-cofilin; Blue, nuclei. Experiments were performed in three independent experiments with three individual replicates. Images shown are representative of the independent experiments. The white arrows indicate actin cytoskeleton rearrangements. Scale bar = 20 μm. Statistically significant differences were determined using one-way ANOVA followed by Tukey *post hoc* test. ^*^p < 0.05.

The LIMK1-cofilin signal cascade can also be directly activated by cAMP/protein kinase A (PKA) in mouse embryonic fibroblasts (Bernstein and Bamburg, 2010). Thus, we investigated whether cAMP/PKA is also involved in gliotoxin-induced actin cytoskeleton rearrangement in lung epithelial cells. First, it was found that the intracellular cAMP content in A549 cells rose significantly after short-time gliotoxin stimulation and reached a peak around 60 min post-stimulation (Figure 4A). Meanwhile, the phosphorylation level of PKA_α/γ_, but not that of PKA_β_, markedly increased in the first 30 min of stimulation and continued to rise up to 90 min (Figure 4B). Further, pretreatment with pCPT-cAMP, a specific agonist of PKA, significantly stimulated the phosphorylation of cofilin and LIMK1 to levels similar to those induced by gliotoxin (Figure 4C). Surprisingly, pretreatment with pCPT-cAMP did not further enhance gliotoxin-induced cofilin and LIMK1 phosphorylation (Figure 4C). In contrast, pretreatment with Rp-cAMP, a specific antagonist of PKA, markedly inhibited gliotoxin-induced phosphorylation of PKA, cofilin, and LIMK1 (Figure 4C). Additional microscopy experiments showed that pCPT-cAMP induced F-actin and p-cofilin accumulation at the cell cortex similar to gliotoxin, but did not enhance the effect induced by gliotoxin (Figure 4D), while Rp-cAMP blocked the actin cytoskeleton rearrangement and cofilin distribution induced by gliotoxin and had no effect on gliotoxin-unstimulated cells. These results suggested that cAMP and PKA are also critical mediators of gliotoxin-induced actin cytoskeleton rearrangement by controlling the LIMK1/cofilin cascade in lung epithelial cells.

### Cdc42, RhoA, and cAMP/PKA Modulate Gliotoxin-Induced Internalization of *A. fumigatus* Into Lung Epithelial Cells

**FIGURE 5:**
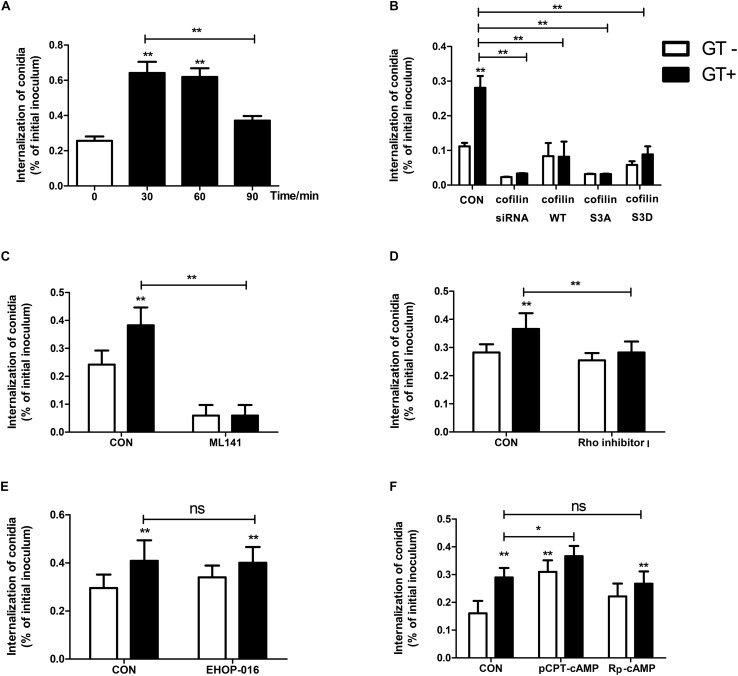
Cdc42, RhoA, and cAMP/PKA modulate gliotoxin-induced internalization of *A. fumigatus* into lung epithelial cells. Prior to inoculation with *A. fumigatus* conidia, A549 cells were treated with gliotoxin for the indicated periods **(A)**, or transfected with siRNA targeting cofilin or plasmid encoding wild-type cofilin, cofilin S3A, or cofilin S3D plasmids **(B)**, or pretreated with 50 μM ML141 (Cdc42 inhibitor) **(C)**, or pretreated with 0.5 μg/ml Rho inhibitor I (RhoA inhibitor) for 1 h **(D)**, or pretreated with 2 μM EHOP-016 (Rac1 inhibitor) for 24 h **(E)**, or pretreated with 100 μM pCPT-cAMP (PKA agonist) or 100 μM Rp-cAMP (PKA antagonist) for 30 min **(F)**, followed by gliotoxin treatment for 30 min. All cells were inoculated with *A. fumigatus* conidia at a MOI of 20 for 6 h. The internalization was assessed by the nystatin protection method. Experiments were performed in three independent experiments with three individual replicates. Statistically significant differences were determined using one-way ANOVA followed by Tukey *post hoc* test or by Dunnett *post hoc* test. ^*^p < 0.05, ^∗∗^*p* < 0.01.

We previously reported that gliotoxin promotes *A. fumigatus* internalization into human lung epithelial cells (Jia et al., 2014). Interestingly, in the current study, A549 cells pretreated with gliotoxin for approximately 30 min and 60 min had the most significant increase in internalization of *A. fumigatus* conidia as compared to untreated cells (Figure 5A). The internalization in A549 cells decreased with the prolongation of gliotoxin treatment. Gliotoxin treatment and internalization into A549 cells seemed not to be simply linearly related. Next, we used two cytoskeleton inhibitors, latrunculin A (blocking the polymerization of F-actin) and cucurbitacin E (inhibitor of F-actin depolymerization) to interfere with gliotoxin-induced actin cytoskeleton rearrangement. Both the inhibitors reduced *A. fumigatus* internalization in a dose-dependent manner (Supplementary Figures S7A,B), without affecting cell viability (Supplementary Figures S7C,D).

Further, we evaluated whether a change in cofilin expression or activity influences *A. fumigatus* internalization into lung epithelial cells. The expression of cofilin in lung epithelial cells transfected with siRNA targeting cofilin or plasmids encoding wild-type cofilin, non-phosphorylatable S3A cofilin, or phosphomimic S3D cofilin was evaluated by western blotting (Supplementary Figure S8). As demonstrated in Figure 5B, cofilin silencing significantly blocked the basal and gliotoxin-promoted internalization of *A. fumigatus* in A549 cells. Intriguingly, a similar inhibitory effect was found in A549 cells overexpressing wild-type cofilin, non-phosphorylatable S3A cofilin, or phosphomimic S3D cofilin. These results suggested that regular cofilin activity (phosphorylation level) might be indispensable in gliotoxin-induced internalization of *A. fumigatus* conidia.

Further, it was interesting to analyze the potential role of small GTPases and cAMP/PKA in gliotoxin-induced internalization of *A. fumigatus*. As shown in Figure 5C, both basal and gliotoxin-promoted internalization of *A. fumigatus* into lung epithelial cells were markedly decreased after pretreatment with a specific inhibitor of Cdc42, ML-141. Comparatively, Rho inhibitor I had no effect on basal internalization; however, it could block gliotoxin-induced conidial internalization (Figure 5D). In contrast, the specific inhibitor of Rac1, EHOP-016, did not affect basal and gliotoxin-induced internalization (Figure 5E), which was in line with its effect on gliotoxin-induced cofilin phosphorylation and actin cytoskeleton. Moreover, the PKA agonist pCPT-cAMP significantly promoted *A. fumigatus* internalization and showed a synergistic effect with gliotoxin on *A. fumigatus* internalization; however, surprisingly, the PKA antagonist Rp-cAMP did not suppress gliotoxin-induced internalization (Figure 5F). These results showed that cAMP/PKA might participate in *A. fumigatus* internalization into lung epithelial cells; however, both Cdc42 and RhoA play the major roles in modulating this process.

### Exogenous Gliotoxin Promotes *A. fumigatus* Invasion in Lung Tissues in Mice Immunosuppressed With Hydrocortisone Acetate

**FIGURE 6:**
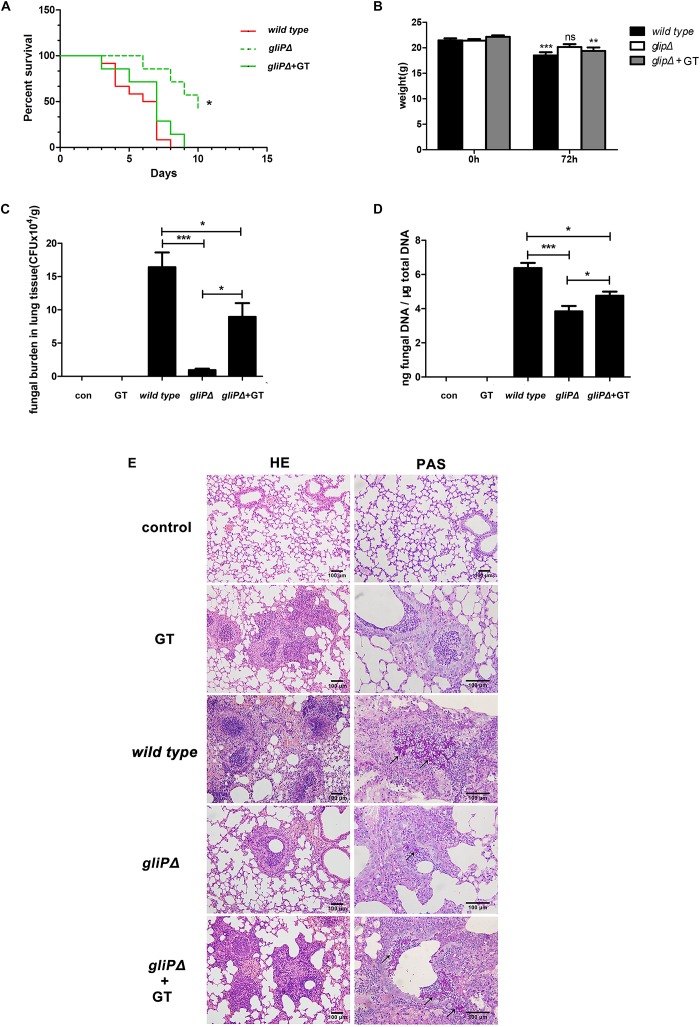
Exogenous gliotoxin promotes invasion of *A. fumigatus* into lung tissue in a mouse model of invasive aspergillosis. C57BL/6 mice were immunosuppressed with hydrocortisone acetate and inoculated intranasally with conidia of *A. fumigatus* wild-type B5233, *gliPΔ*, or *gliPΔ* plus exogenous 50 ng/ml gliotoxin. For survival analysis **(A)** and weight change analysis **(B)**, mice were weighed every 24 h from the day of infection and were visually inspected twice daily. Mice were sacrificed at 72 h post-infection to measure the fungal burden in the lung tissue by counting CFUs **(C)** or by RT-PCR method **(D)**. **(E)** Lung tissues were stained with HE and PAS, and observed by light microscopy (Olympus BX51) at a magnification of 200× or 400×. Black arrows indicate hyphae (red). Experiments were performed in three independent experiments with three individual replicates. Statistically significant differences were determined using one-way ANOVA followed by Tukey *post hoc* test. ^*^p < 0.05, ^∗∗^*p* < 0.01. Statistically significant difference were determined using unpaired Student’s *t*-tests with a 95% confidence interval, using GraphPad Prism software. ^∗∗∗^*p* < 0.001.

In our previous study, gliotoxin was able to promote *A. fumigatus* internalization into lung epithelial cells at a low concentration of 50 ng/ml *in vitro* (Jia et al., 2014), which is much lower than 200 ng/ml used in previous studies (Stanzani et al., 2005; Kupfahl et al., 2006a, 2006b), it was interesting to test whether gliotoxin promotes *A. fumigatus* invasion into lung epithelium *in vivo*. Ten to twelve mice that were immunosuppressed with hydrocortisone acetate were infected with each strain of *A. fumigatus* (wild-type *B5233*, deletion of the peptide synthetase gliP type *gliPΔ*, or *gliPΔ* plus exogenous 50 ng/ml gliotoxin). The survival rate of the *gliPΔ* mutant infected mice was significantly higher than that of wild-type *B5233* infected mice, and exogenous gliotoxin could reduce the survival of mice infected with the *gliPΔ* mutant with statistical significance (Figure 6A). The mice infected with wild-type *B5233* or *glipΔ* mutant strain plus exogenous gliotoxin, but not those infected with the *gliPΔ* mutant strain showed a significant decline in body weight at 3 days post-infection (Figure 6B). Furthermore, exogenous addition of gliotoxin significantly enhanced the pulmonary fungal burden of mice (Figures 6C,D). Pathological staining showed a stronger inflammatory response and more severe invasion of hyphae (black arrow) in the lung tissue, causing tissue necrosis, in mice infected with wild-type *B5233* than in the other two mouse groups. In lung tissues of mice infected with *gliPΔ* alone, the fungus was mainly located in the bronchia, accompanied with an inflammatory response in the peripheral lung tissue. In comparison, infection with *gliPΔ* plus exogenous gliotoxin caused stronger inflammation and a large number of bronchial epithelial shedding, and in some instances even invasion into the lung tissue (Figure 6E). These results suggested that exogenous gliotoxin promoted *A. fumigatus* invasion in lung tissues in the mouse model of invasive aspergillosis immunosuppressed with hydrocortisone acetate in the initial phase of infection.

## Discussion

In present study, it was demonstrated for the first time that gliotoxin was able to induce cofilin phosphorylation and to strongly modulate the actin cytoskeleton dynamic in lung epithelial cells. Interestingly, Cdc42, RhoA, and cAMP/PKA signaling were all involved in gliotoxin-induced actin cytoskeleton rearrangement through LIMK1-cofilin signaling, consequently promoting *A. fumigatus* internalization into A549 cells. These results clarified the role of small GTPases and cofilin under gliotoxin stimulation, which has not been reported before.

An interesting finding in this study was that gliotoxin-induced actin dynamics and internalization of *A. fumigatus* were blocked significantly when the intracellular cofilin phosphorylation cycle was disturbed. It has been demonstrated that the internalization or phagocytosis into host cells requires the cooperation of two forces, one driving the extension of phagocytic cup and the other pulling particles into cells (Herant et al., 2011). This deliberate regulation of *A. fumigatus* internalization by cyclic cofilin phosphorylation is quite similar to the invasion mechanisms of other pathogenic microbes (Chen et al., 2003; Pizarro-Cerda et al., 2004; Yoder et al., 2008; Bernstein and Bamburg, 2010; Xiang et al., 2012; Mizuno, 2013; Zheng et al., 2014). *Listerial* entry into epithelial cells was also markedly interrupted by either increase or defect of cofilin activity, which was attributed to the requirement of the cofilin-involved cooperation of the above-mentioned two forces (Bierne et al., 2001). In addition, during the interaction between *Candida albicans* and mammalian cells, prostaglandin E2 activates cofilin via PTEN to inhibit the formation of actin filaments and the phagocytosis of *C. albicans* by macrophages (Serezani et al., 2012). Our data provided novel evidence that cofilin phosphorylation might be the key intracellular regulating point during phagocytosis as well as in internalization.

As a putative immunosuppressive agent, gliotoxin from *A. fumigatus* inhibits phagocytosis by macrophages and neutrophils. Gliotoxin inhibits phagocytosis via the arachidonic acid signaling pathway in neutrophils (Comera et al., 2007) and by suppressing the production of phosphatidylinositol 3,4,5-trisphosphate in macrophages (Schlam et al., 2016). In contrast, gliotoxin promoted *A. fumigatus* internalization into lung epithelial cells. This difference was not associated with the concentration of gliotoxin we used, as our previous studies showed that 50 ng/ml gliotoxin was also able to inhibit phagocytosis of *A. fumigatus* by macrophages (Jia et al., 2014). Thus, it can be deduced that these two cell types might employ distinct mechanisms in response to gliotoxin, which warrants further exploration in future.

It has been reported that gliotoxin-induced actin cytoskeleton rearrangement is cAMP-dependent in human neutrophils (Comera et al., 2007), but not in macrophages (Schlam et al., 2016), and cAMP/PKA activates LIMK1 directly in mouse embryonic fibroblasts (Bernstein and Bamburg, 2010). Here, it was demonstrated that gliotoxin was able to increase the concentration of intracellular cAMP and to activate PKA; cAMP/PKA signaling regulated gliotoxin-induced LIMK1 and cofilin phosphorylation as well as actin cytoskeleton rearrangement in lung epithelial cells. However, antagonist of PKA could not substantially block gliotoxin-induced internalization of *A. fumigatus* into lung epithelial cells, which might be due to simultaneous gliotoxin-induced strong activation of Cdc42 (data not shown), which could restore the inhibitory effect of the PKA antagonist. In addition, agonist of PKA could not further enhance gliotoxin-induced phosphorylation of cofilin or actin cytoskeleton rearrangement. It seems that in epithelial cells Cdc42 might play relatively predominant roles compared to cAMP/PKA in mediating the gliotoxin-induced cofilin phosphorylation and actin cytoskeleton dynamic signaling.

As a member of the epipolythiodioxopiperazine family of fungal toxins, gliotoxin may function like its analog, sporidesmin to inactivate human glutaredoxin in a time- and concentration-dependent manner (Srinivasan et al., 2006), which might subsequently prohibit the S-glutathionylation and inactivation of Rac1 (Han et al., 2016). Concerning the little effect of Rac1 on gliotoxin-induced actin cytoskeleton and conidial internalization in A549 cells, it seemed not likely that gliotoxin-depleted intracellular glutathione stores plays some role in this actin-based uptake through Rac1. Certainly, since gliotoxin can inhibit the activation of NADPH oxidase which has many cross-talk with actin dynamic (Acevedo and Gonzalez-Billault, 2018), the link between actin-based conidial uptake and gliotoxin-induced redox status change is also an interesting issue to be further investigated.

In addition, novel evidence for the important role of gliotoxin in the development of *A. fumigatus* infection was obtained in this study. In previous studies, the promoting effect of gliotoxin on *A. fumigatus* infection in immunosuppressed mice was only demonstrated by comparing the wild-type strain with gliotoxin biosynthetic gene deletion mutants (Cramer et al., 2006; Spikes et al., 2008; Kwon-Chung and Sugui, 2009). In the present study, based on our findings that gliotoxin promotes the conidia internalization into lung epithelial cells *in vitro*, the direct administration of gliotoxin together with a gliotoxin biosynthetic gene deletion mutant was used in mice for the first time. The increase in fungal burden in lung tissue, obvious lung epithelial decay, and accumulation of conidia and hyphae in alveoli was observed. A small phenomena was noted that the trend of Figures 6C,D are similar, but for fungal DNA, there was a much higher value for the *gliPΔ* mutant compared to the CFUs. This might be attributed to the detection of DNA from the dead or dying conidia or hyphae of *A. fumigatus* whereas the CFU value was only from the culture of living microorganism. Considering that the internalization of *A. fumigatus* into lung alveolar epithelial cells has been demonstrated to be key to infection development (Filler and Sheppard, 2006), it is interesting to speculate that the increased internalization of conidia might be one of the reasons for the positive impact of gliotoxin on *A. fumigatus* infection. Gliotoxin exists not only in dust, bulk samples, and air and ventilation systems of infested buildings, around 40 ng/cm^2^ (Nieminen et al., 2002), but also in the bronchoalveolar lavage fluid of patients suffering from cystic fibrosis (Coughlan et al., 2012) and in serum of invasive aspergillosis patients at rather high concentrations (166–785 ng/ml) (Lewis et al., 2005). Therefore, it seems physiologically feasible that a low amount of gliotoxin from either moldy environments or lung secretions may induce cofilin phosphorylation to help *A. fumigatus* conidial internalization in lung epithelial cells and consequently, to evade host immune attack and establish the initial step of infection, especially in people who have immunologic deficiency. However, we cannot attribute the positive impact of gliotoxin on *A. fumigatus* infection exclusively to a different internalization of conidia, because of the complicated and uncontrollable physiological environment *in vivo*, and roles of other cells, such as macrophages or neutrophils, in response to gliotoxin cannot be excluded. Some more elegant techniques for studying the interaction between *A. fumigatus* and lung epithelial cells specifically *in vivo* are expected in future.

## Conclusion

**FIGURE 7:**
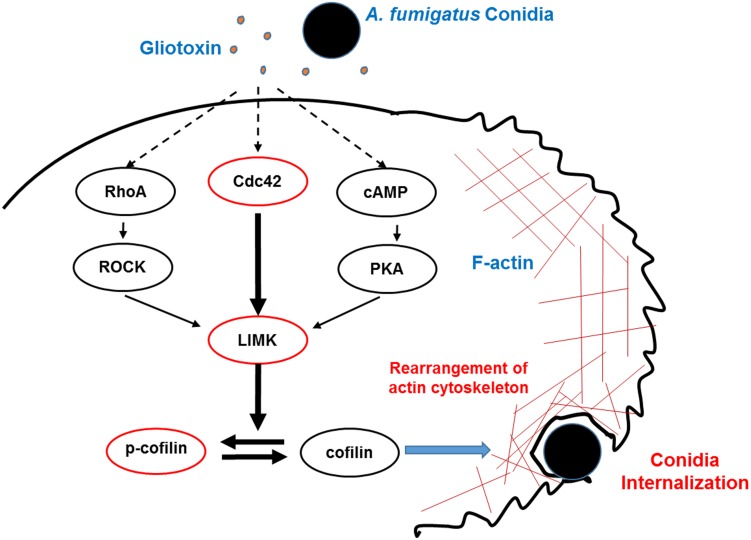
Model of phosphorylation of cofilin and actin cytoskeleton rearrangement induced by gliotoxin during *A. fumigatus* internalization into lung epithelial cells. During the interaction between *A. fumigatus* conidia and lung epithelial cells, gliotoxin, at a rather low concentration, might induce cofilin phosphorylation mainly through Cdc42 signaling, and in addition, through RhoA activation and increases in cAMP and PKA activation, which all lead to LIMK1 activation and phosphorylation of cofilin, which subsequently modulates actin cytoskeleton rearrangement and facilitates the internalization of conidia into host cells. Dashed lines indicate signal pathway interactions hypothesized to occur in A549 cells.

In conclusion, the present study elucidated for the first time that gliotoxin, an essential toxin from *A. fumigatus*, stimulates cofilin phosphorylation through the Cdc42/RhoA-LIMK1 and cAMP/PKA-LIMK1 signaling pathways, and consequently, modulates actin cytoskeleton rearrangement to facilitate *A. fumigatus* internalization into lung epithelial cells (Figure 7).

## Ethics Statement

This study was carried out in accordance with the Laboratory Animal Welfare and Ethics Committee (IACUC-13-2016-002) and was conducted in accordance with relevant guidelines and regulations for laboratory animals.

## Author Contributions

CZ, FC, and LH planned the experiments, analyzed the data, and wrote the manuscript. XL, XH, YH, and XS performed the experiments. YC and YS contributed reagents or other essential material.

## Conflict of Interest Statement

The authors declare that the research was conducted in the absence of any commercial or financial relationships that could be construed as a potential conflict of interest.

## Supplementary Material

FIGURE S1 Gliotoxin at 50 ng/ml does not induce apoptosis and cell death in A549 lung epithelial cells and Beas2B cells. The cells were treated with 50 ng/ml gliotoxin for the indicated periods. **(A)** Flow cytometry was used to quantify cell apoptosis with the Annexin V-FITC/PI Apoptosis Detection Kit (CW BIOtech, CW2574, China). The proportions of apoptosis **(B)** and cell death **(C)** for indicated times were quantitatively calculated according to the flow-cytometric results. The viability of A549 cells **(D)** and Beas2B cells **(E)** was tested by MTT assay. Experiments were performed in three independent experiments with three individual replicates. Statistically significant differences were determined using one-way ANOVA followed by Tukey *post hoc* test.^*^*p* < 0.05.

FIGURE S2 Actin cytoskeleton rearrangement during the internalization of *A. fumigatus* conidia into lung epithelial cells. Four images for two conidia were captured at different time points. During the internalization of *A. fumigatus* ATCC13073 conidia, a red-labeled actin cap at the initial invasion site into A549 cells (Time 1:33:54, up and left panel, white arrow or Time 3:09:54, up and right panel, white arrow); and later enriched around an internalized conidium and formed a red-labeled ring (Time 1:55:54, down and left panel or Time 3:45:52, down and right panel). Red, F-actin; Green, conidia. Bar = 25 μm.

FIGURE S3 Localization of Cofilin with *A. fumigatus* conidia in lung epithelial cells. When *A. fumigatus* Ku80 conidia internalized into A549 cells (Time 3:46:02, up panel, white arrow or Time 4:46:02, down panel, white arrow), the red conidia and green GFP-cofilin merged together to form a yellow ring around red conidia. Bar = 25 μm.

FIGURE S4 Supplementary evidences on gliotoxin-induced cofilin phosphorylation. **(A)** The expression and phosphorylation level of cofilin in Beas2B cells treated with gliotoxin for the indicated periods. **(B)** The expression and phosphorylation level of cofilin in A549 cells exposed to the culture supernatant of different strains (WT, *glipΔ*, and *glipR*). Experiments were performed in three independent experiments with three individual replicates. Images shown are representative of the independent experiments.

FIGURE S5 Verification of GFP-cofilin expression in A549 cells transfected with different plasmids. A549 cells were transfected with the plasmids encoding GFP-cofilin WT, GFP-cofilin S3A, or GFP-cofilin S3D for 48 h. Then, the expression of GFP-cofilin and cofilin was verified by western blotting **(A)** or fluorescence detection **(B)**. Experiments were performed in three independent experiments with three individual replicates. Images shown are representative of the independent experiments. Scale bar = 100 μm.

FIGURE S6 Viability of A549 cells after treatment with chemical reagents. A549 cells were treated with 0.5 μg/ml Rho inhibitor I for the indicated periods **(A)**, or ML141 at different concentrations for 60 min **(B)**, or EHOP-016 at different concentrations for 24 h **(C)**. Viability was evaluated by MTT assay. Experiments were performed in three independent experiments with three individual replicates. Statistically significant differences were determined using one-way ANOVA followed by Tukey *post hoc* test. ^∗∗^*p* < 0.01, ^*^*p* < 0.05.

FIGURE S7 Effects of actin dynamic inhibitors on the internalization of *A. fumigatus* into A549 cells. A549 cells were pretreated with LatA **(A,C)** or CuE **(B,D)** at different concentrations for 60 min prior to inoculation with *A. fumigatus* conidia. The internalization was assessed by the nystatin protection method and the viability was evaluated by MTT assay. Experiments were performed in three independent experiments with three individual replicates. Statistically significant differences were determined using one-way ANOVA followed by Tukey *post hoc* test. ^*^*p* < 0.05, ^∗∗^*p* < 0.01, ^∗∗∗^*p* < 0.001.

FIGURE S8 Verification of cofilin expression in A549 cells transfected with different plasmids. A549 cells were transfected with cofilin siRNA and the plasmids encoding cofilin WT, cofilin S3A, or cofilin S3D. Then, the expression of cofilin was verified by western blotting. Experiments were performed in three independent experiments with three individual replicates. Images shown are representative of the independent experiments.

MOVIE S1 Video for the process of *A. fumigatus* ATCC13073 conidia internalization into lung epithelial cells. The process of *A. fumigatus* ATCC13073 conidia internalization into lung epithelial cells was monitored by Time-lapse spinning disk confocal microscope and maintained at 37°C and 5% CO_2_ during live-cell imaging. Red, F-actin; Green, conidia. Bar = 25 μm.

MOVIE S2 Video for visualizing the cofilin and *A. fumigatus* conidia internalization in lung epithelial cells. The distribution of cofilin during *A. fumigatus* conidia internalization in lung epithelial cells was monitored by Time-lapse spinning disk confocal microscope and maintained at 37°C and 5% CO_2_ during live-cell imaging. Red, conidia; Green, cofilin. Bar = 25 μm.
