# Supplementary figures and images for: Gliotoxin Induces Cofilin Phosphorylation to Promote Actin Cytoskeleton Dynamics and Internalization of Aspergillus fumigatus Into Type II Human Pneumocyte Cells
Source: Front Microbiol. 2019 Jun 18;10:1345. doi: 10.3389/fmicb.2019.01345 (PMC6591310; doi:10.3389/fmicb.2019.01345)

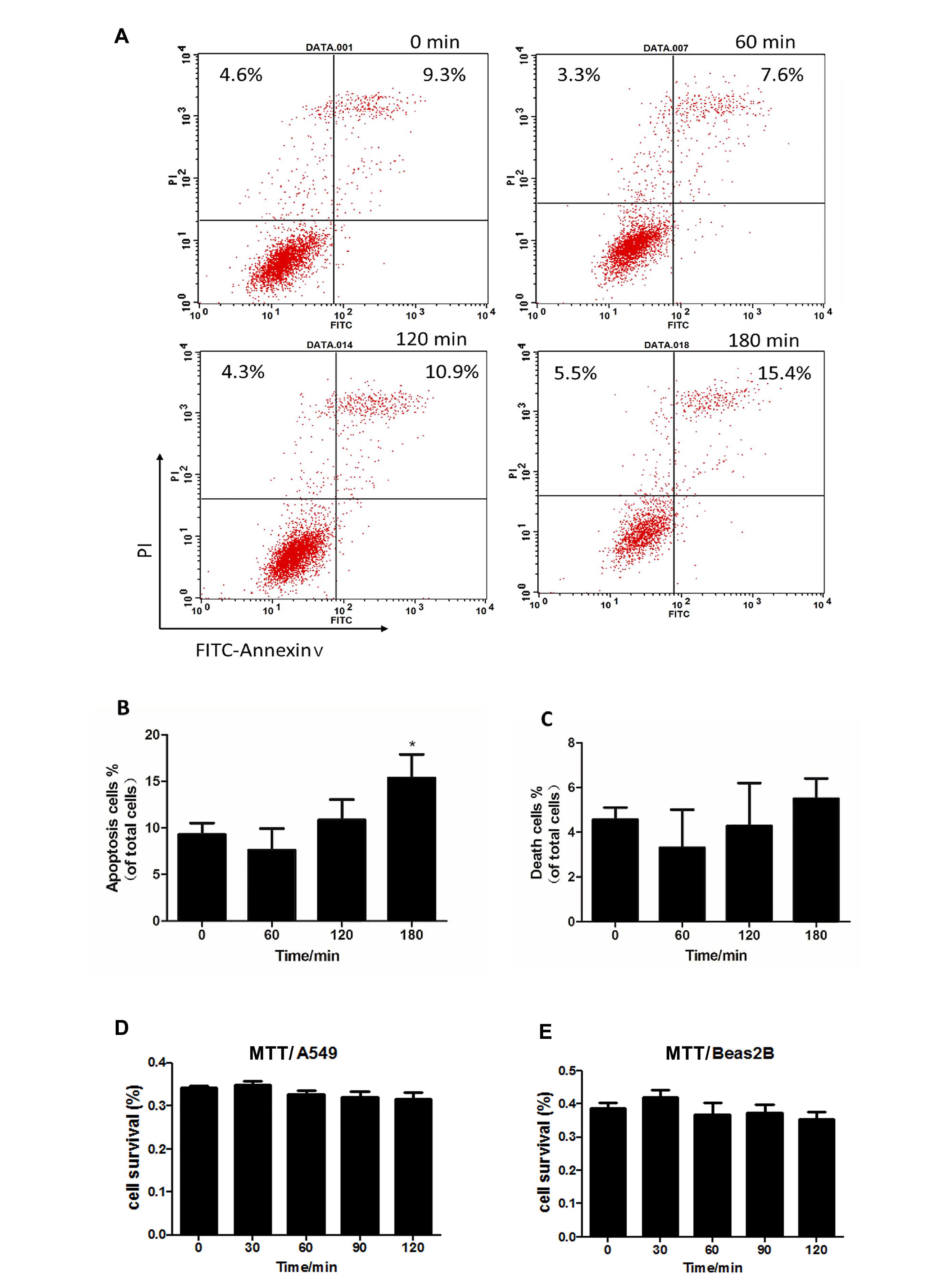

Supplement: FIGURE S1 — Gliotoxin at 50 ng/ml does not induce apoptosis and cell death in A549 lung epithelial cells and Beas2B cells. The cells were treated with 50 ng/ml gliotoxin for the indicated periods. (A) Flow cytometry was used to quantify cell apoptosis with the Annexin V-FITC/PI Apoptosis Detection Kit (CW BIOtech, CW2574, China). The proportions of apoptosis (B) and cell death (C) for indicated times were quantitatively calculated according to the flow-cytometric results. The viability of A549 cells (D) and Beas2B cells (E) was tested by MTT assay. Experiments were performed in three independent experiments with three individual replicates. Statistically significant differences were determined using one-way ANOVA followed by Tukey post hoc test.*p < 0.05. [file Data_Sheet_1.ZIP › Supplemental figures/supplemental figure 1.tif]

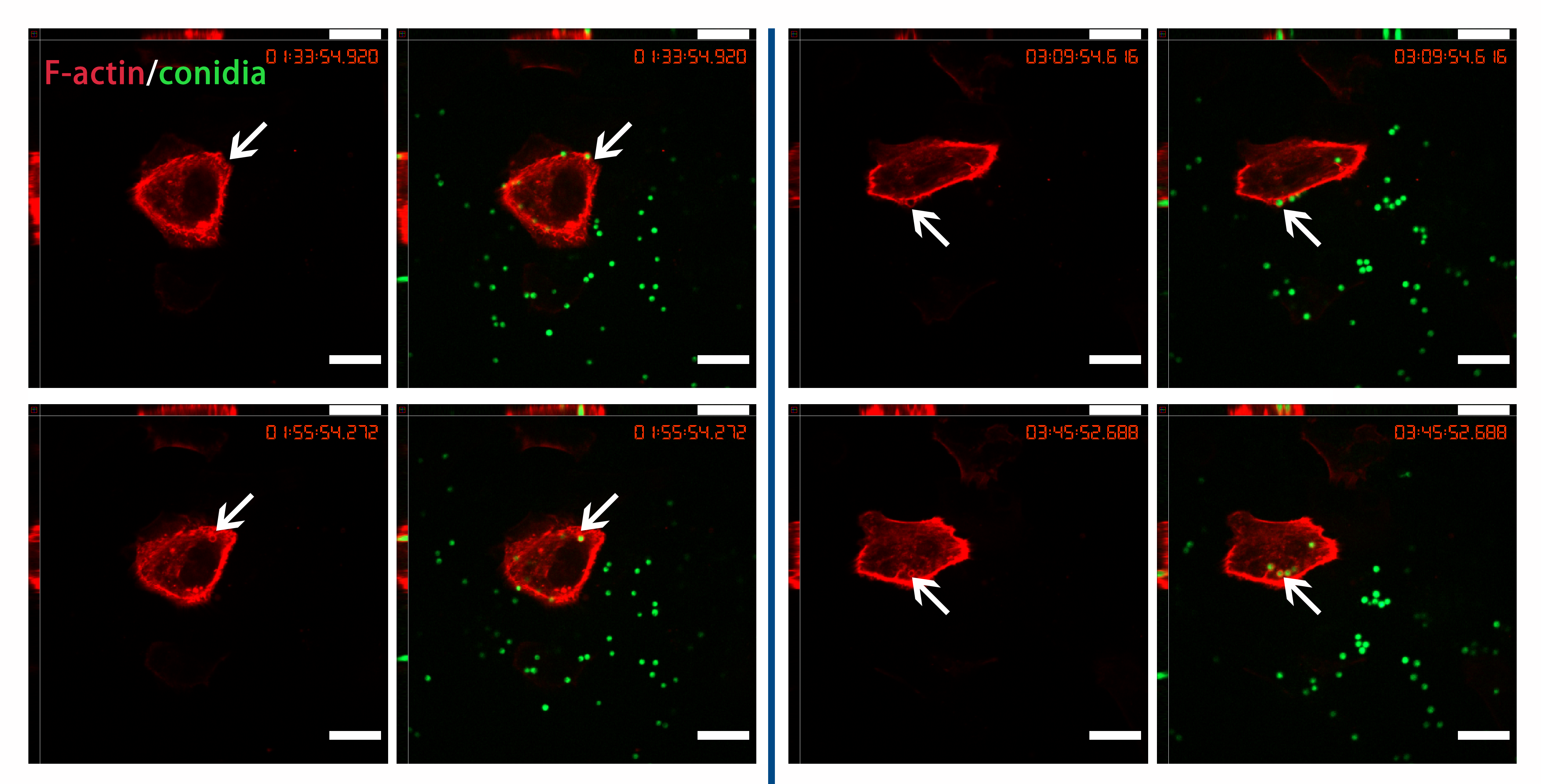

Supplement: FIGURE S1 — Gliotoxin at 50 ng/ml does not induce apoptosis and cell death in A549 lung epithelial cells and Beas2B cells. The cells were treated with 50 ng/ml gliotoxin for the indicated periods. (A) Flow cytometry was used to quantify cell apoptosis with the Annexin V-FITC/PI Apoptosis Detection Kit (CW BIOtech, CW2574, China). The proportions of apoptosis (B) and cell death (C) for indicated times were quantitatively calculated according to the flow-cytometric results. The viability of A549 cells (D) and Beas2B cells (E) was tested by MTT assay. Experiments were performed in three independent experiments with three individual replicates. Statistically significant differences were determined using one-way ANOVA followed by Tukey post hoc test.*p < 0.05. [file Data_Sheet_1.ZIP › Supplemental figures/supplemental figure 2.tif]

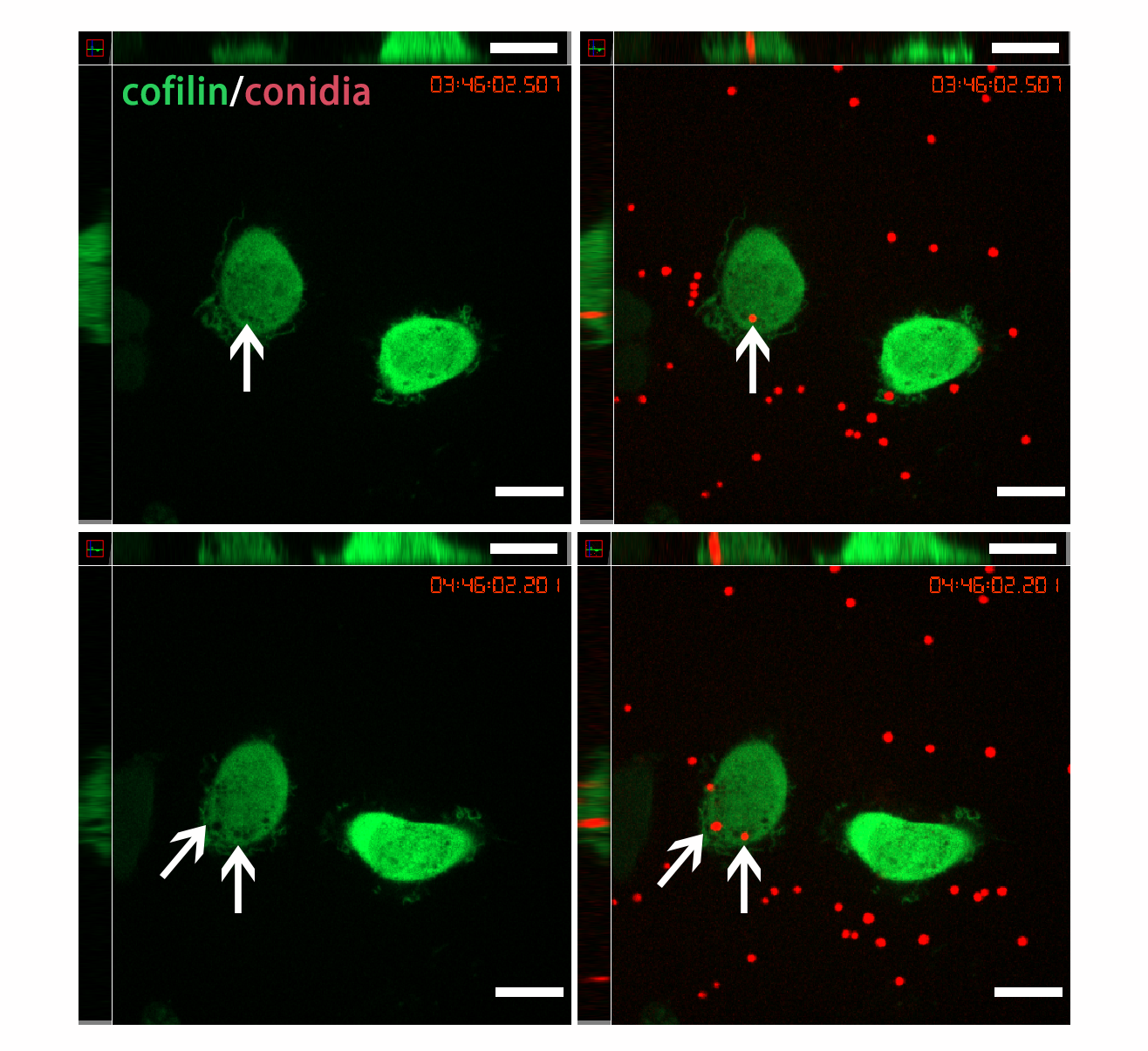

Supplement: FIGURE S1 — Gliotoxin at 50 ng/ml does not induce apoptosis and cell death in A549 lung epithelial cells and Beas2B cells. The cells were treated with 50 ng/ml gliotoxin for the indicated periods. (A) Flow cytometry was used to quantify cell apoptosis with the Annexin V-FITC/PI Apoptosis Detection Kit (CW BIOtech, CW2574, China). The proportions of apoptosis (B) and cell death (C) for indicated times were quantitatively calculated according to the flow-cytometric results. The viability of A549 cells (D) and Beas2B cells (E) was tested by MTT assay. Experiments were performed in three independent experiments with three individual replicates. Statistically significant differences were determined using one-way ANOVA followed by Tukey post hoc test.*p < 0.05. [file Data_Sheet_1.ZIP › Supplemental figures/supplemental figure 3.tif]

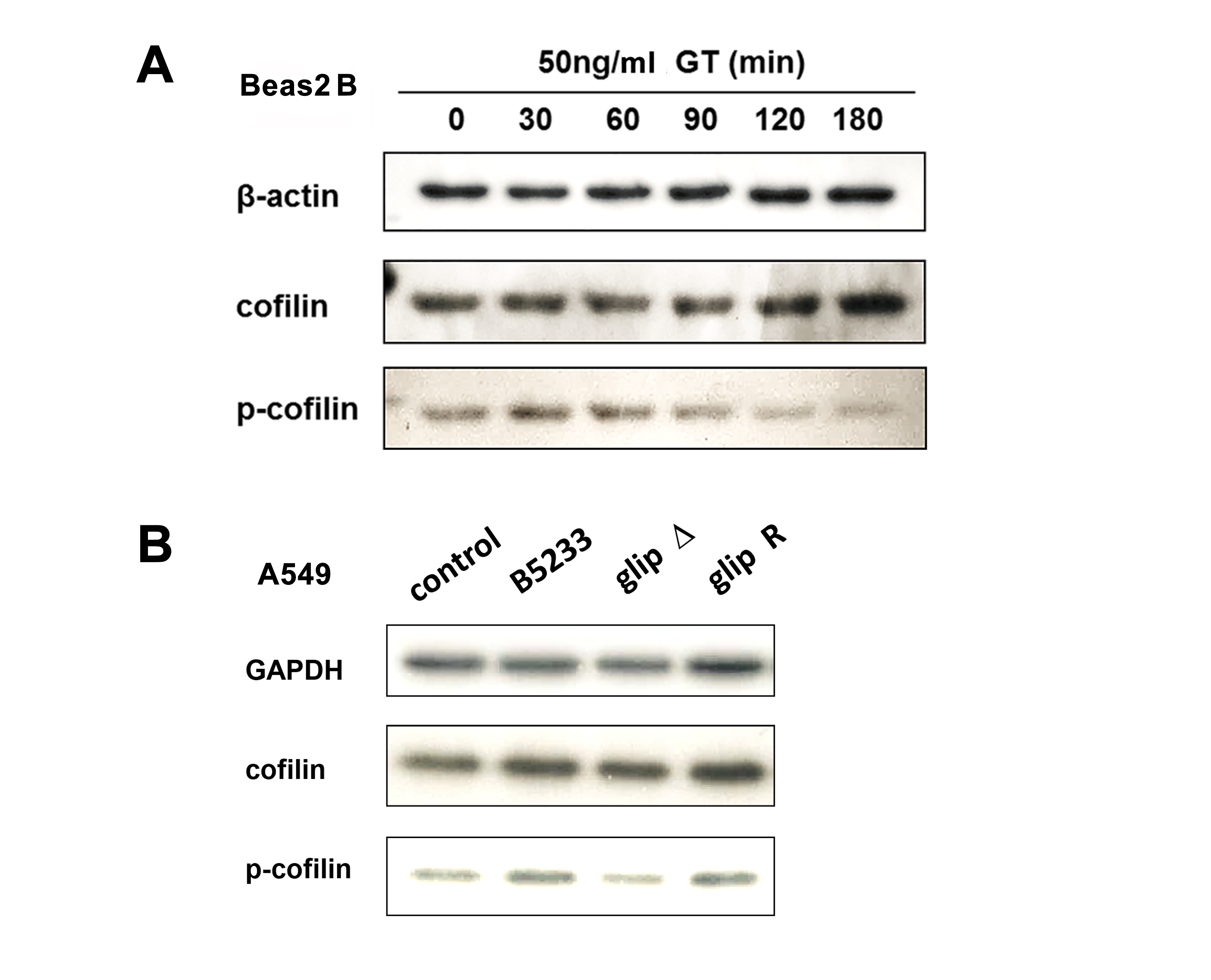

Supplement: FIGURE S1 — Gliotoxin at 50 ng/ml does not induce apoptosis and cell death in A549 lung epithelial cells and Beas2B cells. The cells were treated with 50 ng/ml gliotoxin for the indicated periods. (A) Flow cytometry was used to quantify cell apoptosis with the Annexin V-FITC/PI Apoptosis Detection Kit (CW BIOtech, CW2574, China). The proportions of apoptosis (B) and cell death (C) for indicated times were quantitatively calculated according to the flow-cytometric results. The viability of A549 cells (D) and Beas2B cells (E) was tested by MTT assay. Experiments were performed in three independent experiments with three individual replicates. Statistically significant differences were determined using one-way ANOVA followed by Tukey post hoc test.*p < 0.05. [file Data_Sheet_1.ZIP › Supplemental figures/supplemental figure 4.tif]

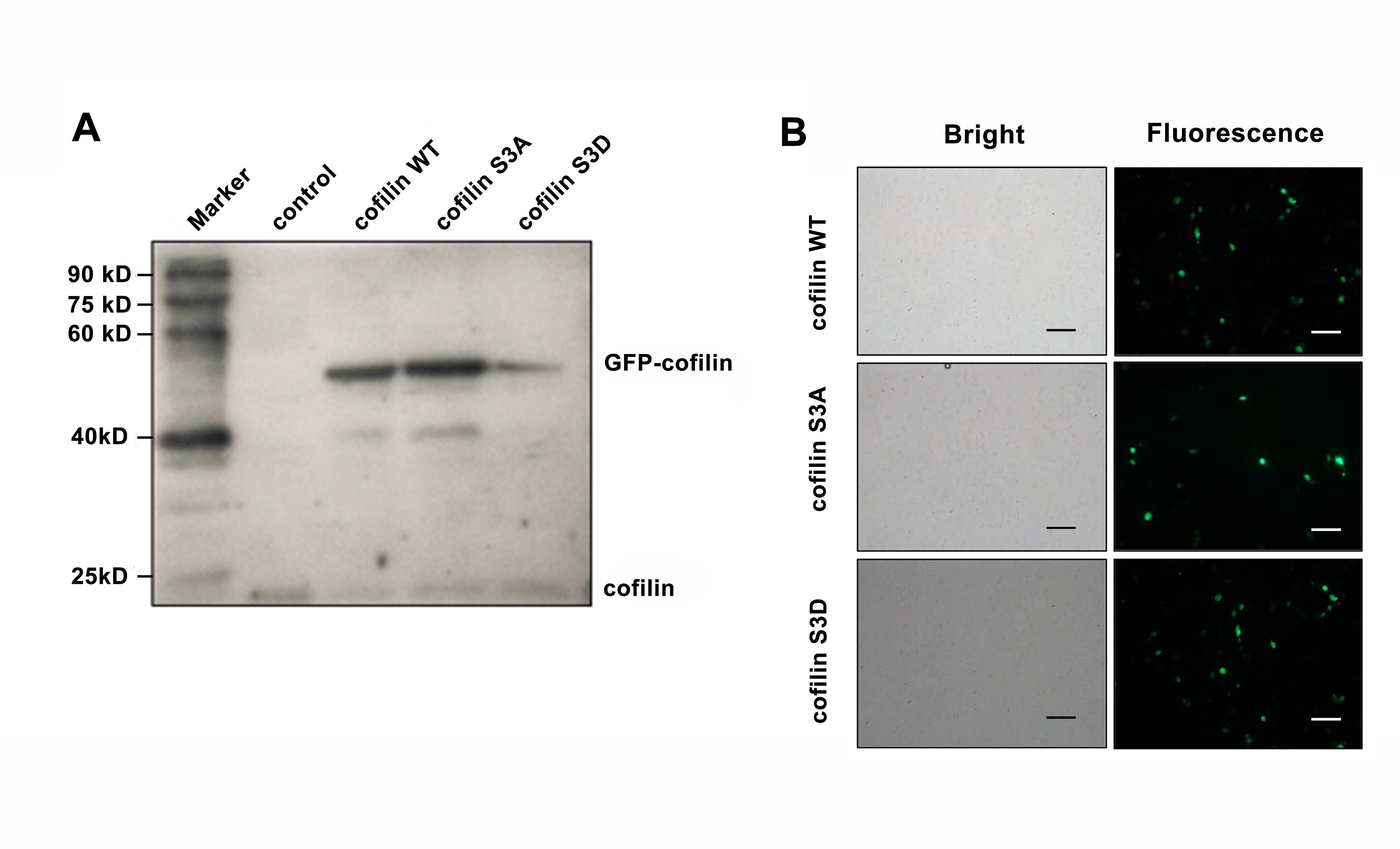

Supplement: FIGURE S1 — Gliotoxin at 50 ng/ml does not induce apoptosis and cell death in A549 lung epithelial cells and Beas2B cells. The cells were treated with 50 ng/ml gliotoxin for the indicated periods. (A) Flow cytometry was used to quantify cell apoptosis with the Annexin V-FITC/PI Apoptosis Detection Kit (CW BIOtech, CW2574, China). The proportions of apoptosis (B) and cell death (C) for indicated times were quantitatively calculated according to the flow-cytometric results. The viability of A549 cells (D) and Beas2B cells (E) was tested by MTT assay. Experiments were performed in three independent experiments with three individual replicates. Statistically significant differences were determined using one-way ANOVA followed by Tukey post hoc test.*p < 0.05. [file Data_Sheet_1.ZIP › Supplemental figures/supplemental figure 5.tif]

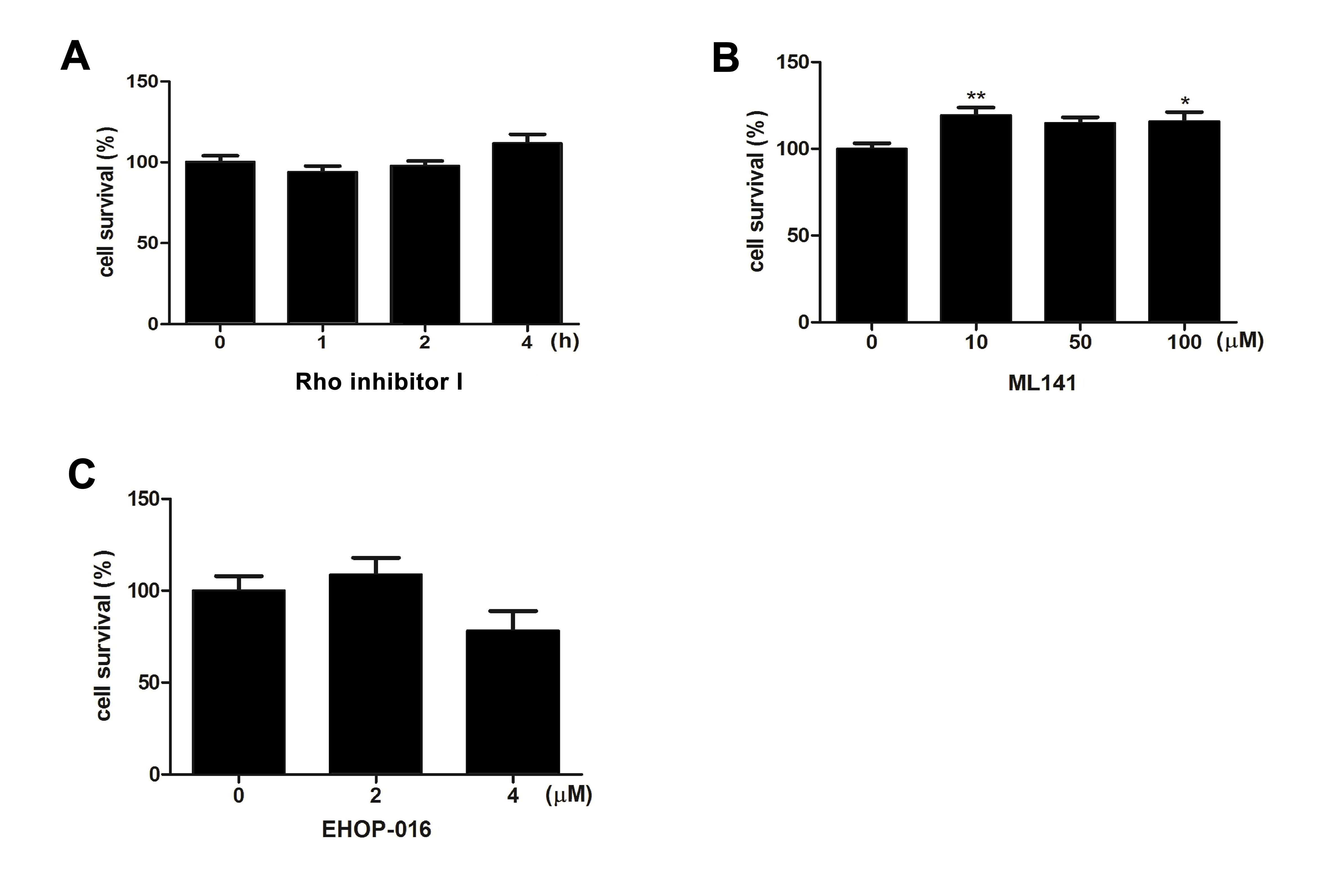

Supplement: FIGURE S1 — Gliotoxin at 50 ng/ml does not induce apoptosis and cell death in A549 lung epithelial cells and Beas2B cells. The cells were treated with 50 ng/ml gliotoxin for the indicated periods. (A) Flow cytometry was used to quantify cell apoptosis with the Annexin V-FITC/PI Apoptosis Detection Kit (CW BIOtech, CW2574, China). The proportions of apoptosis (B) and cell death (C) for indicated times were quantitatively calculated according to the flow-cytometric results. The viability of A549 cells (D) and Beas2B cells (E) was tested by MTT assay. Experiments were performed in three independent experiments with three individual replicates. Statistically significant differences were determined using one-way ANOVA followed by Tukey post hoc test.*p < 0.05. [file Data_Sheet_1.ZIP › Supplemental figures/supplemental figure 6.tif]

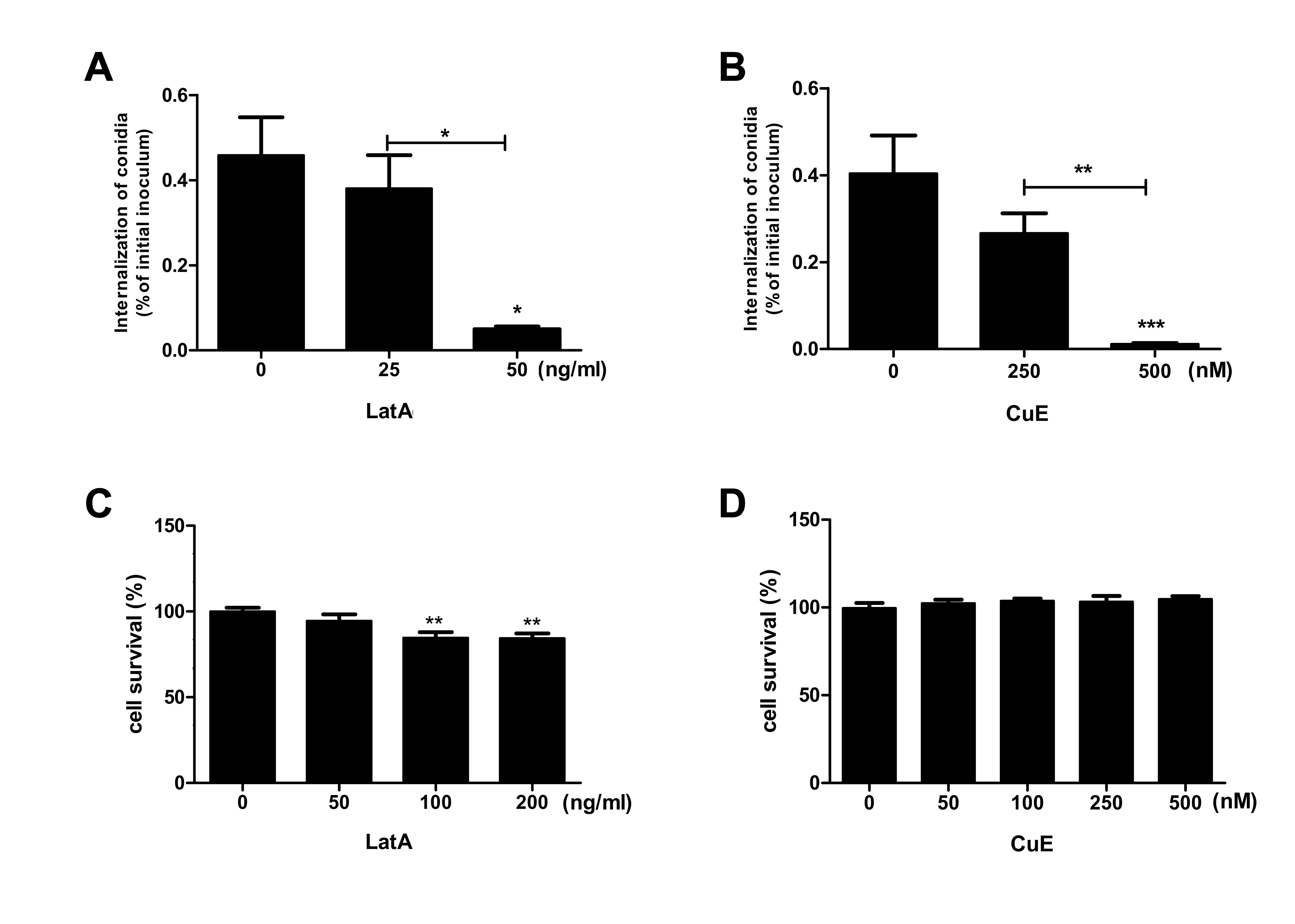

Supplement: FIGURE S1 — Gliotoxin at 50 ng/ml does not induce apoptosis and cell death in A549 lung epithelial cells and Beas2B cells. The cells were treated with 50 ng/ml gliotoxin for the indicated periods. (A) Flow cytometry was used to quantify cell apoptosis with the Annexin V-FITC/PI Apoptosis Detection Kit (CW BIOtech, CW2574, China). The proportions of apoptosis (B) and cell death (C) for indicated times were quantitatively calculated according to the flow-cytometric results. The viability of A549 cells (D) and Beas2B cells (E) was tested by MTT assay. Experiments were performed in three independent experiments with three individual replicates. Statistically significant differences were determined using one-way ANOVA followed by Tukey post hoc test.*p < 0.05. [file Data_Sheet_1.ZIP › Supplemental figures/supplemental figure 7.tif]

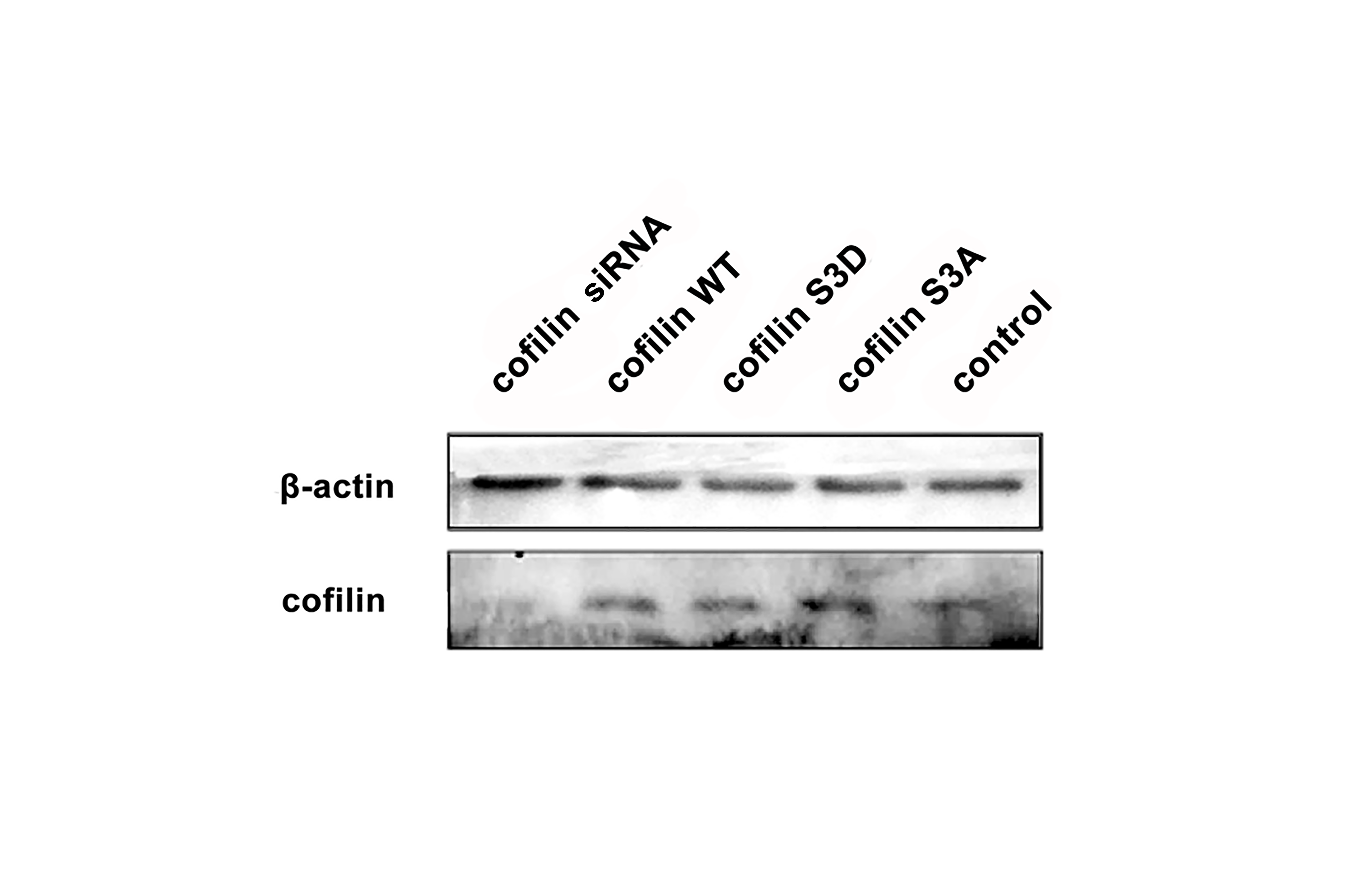

Supplement: FIGURE S1 — Gliotoxin at 50 ng/ml does not induce apoptosis and cell death in A549 lung epithelial cells and Beas2B cells. The cells were treated with 50 ng/ml gliotoxin for the indicated periods. (A) Flow cytometry was used to quantify cell apoptosis with the Annexin V-FITC/PI Apoptosis Detection Kit (CW BIOtech, CW2574, China). The proportions of apoptosis (B) and cell death (C) for indicated times were quantitatively calculated according to the flow-cytometric results. The viability of A549 cells (D) and Beas2B cells (E) was tested by MTT assay. Experiments were performed in three independent experiments with three individual replicates. Statistically significant differences were determined using one-way ANOVA followed by Tukey post hoc test.*p < 0.05. [file Data_Sheet_1.ZIP › Supplemental figures/supplemental figure 8.tif]
